# Welly: a web-tool for visualizing growth curves from microplate data

**DOI:** 10.1093/bioadv/vbaf038

**Published:** 2025-03-04

**Authors:** Felix Meier, Tom Williams, Ian Paulsen

**Affiliations:** School of Natural Sciences, Macquarie University, Sydney 2109, Australia; ARC Centre of Excellence in Synthetic Biology, Sydney 2109, Australia; School of Natural Sciences, Macquarie University, Sydney 2109, Australia; ARC Centre of Excellence in Synthetic Biology, Sydney 2109, Australia; School of Natural Sciences, Macquarie University, Sydney 2109, Australia; ARC Centre of Excellence in Synthetic Biology, Sydney 2109, Australia

## Abstract

**Summary:**

Welly is a web-based tool designed to simplify the visualization and analysis of growth curves from 96- and 384-well plates, addressing the limitations of existing commercial and coding-based solutions. Users can upload plate reader data in CSV or Excel format, easily select sample names and replicates and Welly generates interactive growth curves displaying the mean and standard deviation of triplicates. Additional features include heat map visualizations of maximum values, and downloadable interactive graphs of publication-quality figures and statistics files containing area under curve and max growth rate value of replicates.

**Availability and implementation:**

Welly is freely available at https://synbioexplorer.pythonanywhere.com, providing an easy-to-use interface accessible to all. All the code is publicly available at the github repository https://github.com/SynBioExplorer/Welly under the MIT license. The website will remain freely accessible for at least 2 years post publication, likely longer.

## 1 Introduction

Visualization of growth curves from plate readers is a fundamental task in biotechnological and microbiological laboratories. These experiments often involve collecting optical density readings from microplate readers, typically in 96- or 384-well formats, to monitor microbial growth, metabolic activity, enzyme assays, or other biological processes. Tools to visualize and analyze well data exist, but they either require some coding knowledge of R/Python libraries such as ggplate (Quast 2024), Wellmap (Kundert 2021), AMiGA (Midani *et al.* 2021), and gcplyr (Blazanin 2024), or they are only available commercially (SoftMax Pro, GraphPad Prism, Gen5 Data Analysis). The commercial applications require installation not necessarily suitable to all operating systems with licenses ranging from hundreds to thousands of dollars annually. The code-based solutions are free to use but tend to be harder to access for users with limited coding experience. Another web-based solution, Dashing Growth Curves, offers advanced features for fitting growth models and extracting detailed growth parameters, such as lag time and exponential phase transitions, but it is designed for general microbial datasets without tailored support for well-plate workflows (Reiter and Vorholt 2024). In contrast, Welly focuses on user-friendly visualization and streamlined analysis specifically for 96- and 384-well plate data, with an emphasis on intuitive sample selection, handling, and naming. While some of these programs offer more functionality, they also require extensive time commitment to achieve more sophisticated results.

Due to the technical difficulty and/or cost of existing solutions, many lab users end up manually calculating means, standard deviations, and visualizing using Microsoft Excel or Google Sheets which can be quite time-consuming and visually unappealing. Welly addresses these issues by providing a web-based tool with an easy-to-use interface, producing interactive graphs with publication-level quality within minutes. It does not require installation, coding knowledge, payment, or user registration. Researchers are only requested to cite Welly in any publications resulting from its use. Users can optionally subscribe to our mailing list to receive updates about new features and improvements. This subscription is entirely voluntary and not required to use the platform.

## 2 Features

**Figure 1.**
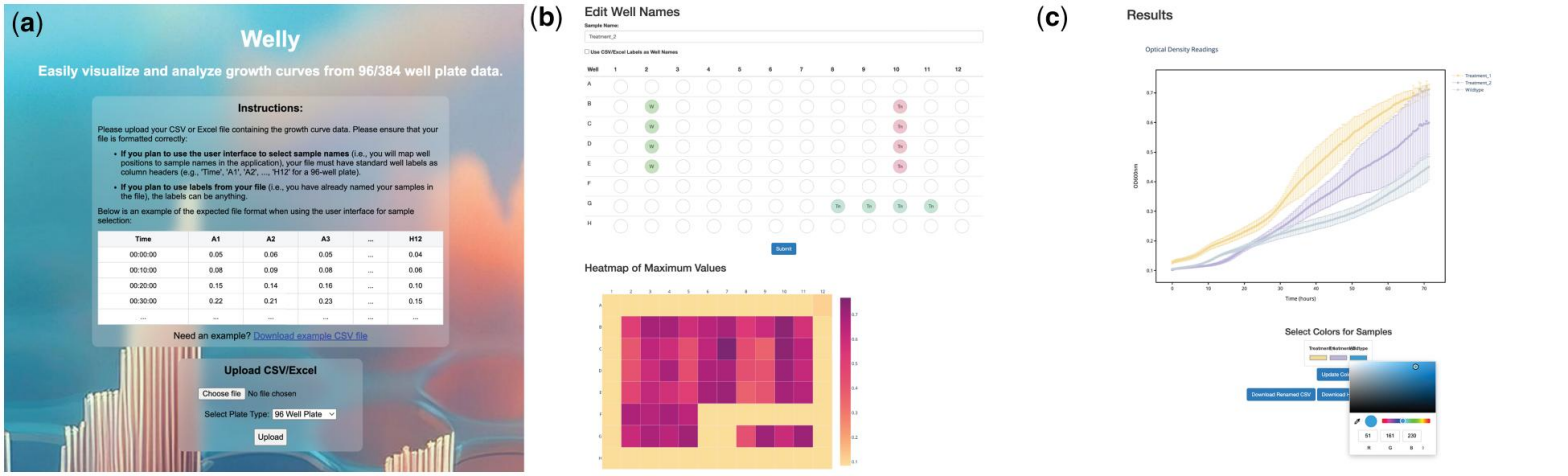
Illustration of Welly workflow. (a) Researchers upload 96 or 384 well plate reader data in Excel or CSV format. (b) Users input their sample name and then drag and drop them onto the 96/384—plate GUI. A heatmap of maximum well values can serve as an orientation to which wells are occupied. Alternatively, users can select the input data values and proceed without sample selection. (c) interactive plotly visualization of sample growth curves displayed as mean ± standard deviation. Plotly graph can be downloaded as an interactive html or static high resolution png file. A file containing statistics such as max growth rate and area under curve analysis for each sample replicate can be downloaded as well.

Workflow (Fig. 1): Researchers are asked to upload a csv or excel file containing a time column and column optical density data from A1 to H12 (96-well) or from A1-P24 (384-well) to an external server. After submission, users see a heatmap of max values per well to get an indication of which wells contain samples and which ones are empty. They can then use the GUI to name and select replicate samples. Alternatively, the column headers of their submitted data can be used as sample input names where the same names get treated as replicates. After submission, Welly produces an interactive growth curve displaying the means and standard deviations of said samples. The graph can be downloaded as a picture or as an interactive html file. Additionally, a downloadable interactive html file displays a heat map showing the max OD values per well, bar graphs showing max growth rate ± standard deviation and area under curve ± standard deviation. Users have the option to customize sample colors. A comprehensive step-by-step guide for using Welly is available in the supplementary material, providing detailed instructions on data upload, sample selection, and interpretation of visualization outputs.

Software stack: Welly’s software stack primarily uses Python and Flask for backend processing, managing routes, file uploads, and rendering dynamic content. The frontend is powered by HTML templates with Jinja2, enabling data-driven elements and seamless integration with user inputs. Bootstrap 3.3.7 ensures responsive design and a clean, user-friendly interface across devices. For visualizing data, Welly leverages Plotly to create interactive graphs, such as growth curves and heatmaps, while JavaScript enables client-side interactions, including real-time modifications to well plates. The service is hosted on pythonanywhere.com, a reliable and scalable cloud-based environment. While a purely browser-based implementation could achieve similar functionality, the server-based architecture of Welly provides critical advantages. By centralizing data processing and visualization, it ensures consistent performance across all major browsers and devices, mitigating potential compatibility issues with diverse user environments. Additionally, this approach supports future extensibility and support to ensure that Welly remains a sustainable and evolving solution for its users.

## 3 Conclusion and discussion

### 3.1 Current impact and contributions

Welly provides a streamlined, accessible, and efficient solution for visualizing growth curves from 96- and 384-well plate data. It offers a user-friendly alternative to both commercial software and coding-based approaches, making it suitable for experienced researchers as well as students with limited technical expertise. By eliminating the need for coding knowledge, costly software licenses, or complex installations, Welly allows users to focus on their analysis, significantly reducing the time spent on data processing and visualization.

### 3.2 Design decisions and current limitations

While Welly prioritizes accessibility and ease of use, this design philosophy necessarily involves trade-offs regarding specialized analytical capabilities. The current version focuses on core functionality, deliberately omitting features such as curve fitting, statistical significance testing, and automated blank subtraction to maintain a straightforward user experience. For researchers requiring these capabilities, data can be easily exported to specialized statistical software, maintaining workflow flexibility while preserving Welly’s streamlined interface. Graph customization in Welly currently prioritizes essential features over comprehensive options. The tool generates publication-ready interactive plots with customizable colors and labels, though advanced options such as font selection and alternative graph types are not yet implemented. This approach aligns with our goal of providing immediate utility to researchers while maintaining a user-centered intuitive interface.

### 3.3 Market position and significance

Welly addresses a crucial gap in the laboratory software ecosystem by providing an intuitive, web-based solution for data visualization, particularly valuable for researchers who lack access to expensive software or coding expertise. Its open-access platform ensures seamless compatibility across all common operating systems and browsers, eliminating traditional barriers such as software installations and licensing costs. For those prioritizing speed, efficiency, and ease of use, Welly delivers a highly compelling alternative to both commercial solutions and complex coding-based approaches. The platform has been extensively tested for compatibility with output formats from major plate reader manufacturers. The open-source nature of the project enables community contributions, ensuring sustainable long-term development and potential implementation of additional features.

### 3.4 Concluding statement

Welly successfully addresses an underserved market niche by providing an accessible, efficient platform for growth curve visualization without the complexity of installation or licensing barriers. While it may not currently offer advanced statistical analysis or deep customization options, it excels in its primary mission: enabling fast, straightforward data visualization for laboratory researchers. Through continued development and community engagement, Welly is positioned to evolve into an even more valuable resource for the scientific community, maintaining its balance between simplicity and capability.

## Supplementary Material

vbaf038_Supplementary_Data

## Data Availability

Welly is freely available at https://synbioexplorer.pythonanywhere.com, providing an easy-to-use interface accessible to all. All the code is publicly available at the github repository https://github.com/SynBioExplorer/Welly under the MIT license. The website will remain freely accessible for at least 2 years post publication, likely longer.
