# Supplementary material for: Welly: a web-tool for visualizing growth curves from microplate data
Source: Bioinform Adv. 2025 Mar 4;5(1):vbaf038. doi: 10.1093/bioadv/vbaf038 (PMC11908640; doi:10.1093/bioadv/vbaf038)
Supplement: vbaf038_Supplementary_Data [file vbaf038_supplementary_data.pdf]

Welly User Guide

Features Overview

- Upload and Preview Data - Upload CSV or Excel files for analysis.
- Interactive Well Plate Editor - Assign sample names and select replicates directly on a visual grid.
- Data Analysis - Calculate means and standard deviations for replicates.
- Data Visualization - Generate growth curves and summary statistics.
- Report Generation - Download detailed HTML reports.

Step-by-Step Guide

Step 1: Upload Data

1. Navigate to the home page of Welly.

2. Prepare your data file according to the required format:

- The first column must be labeled 'Time' with values like hh:mm:ss.

- Remaining columns should correspond to well positions (e.g., A1, A2, ..., P24 for 384-well plates).

3. On the Upload CSV/Excel section:

- Select the file format and plate type.

- Click 'Upload' to proceed.

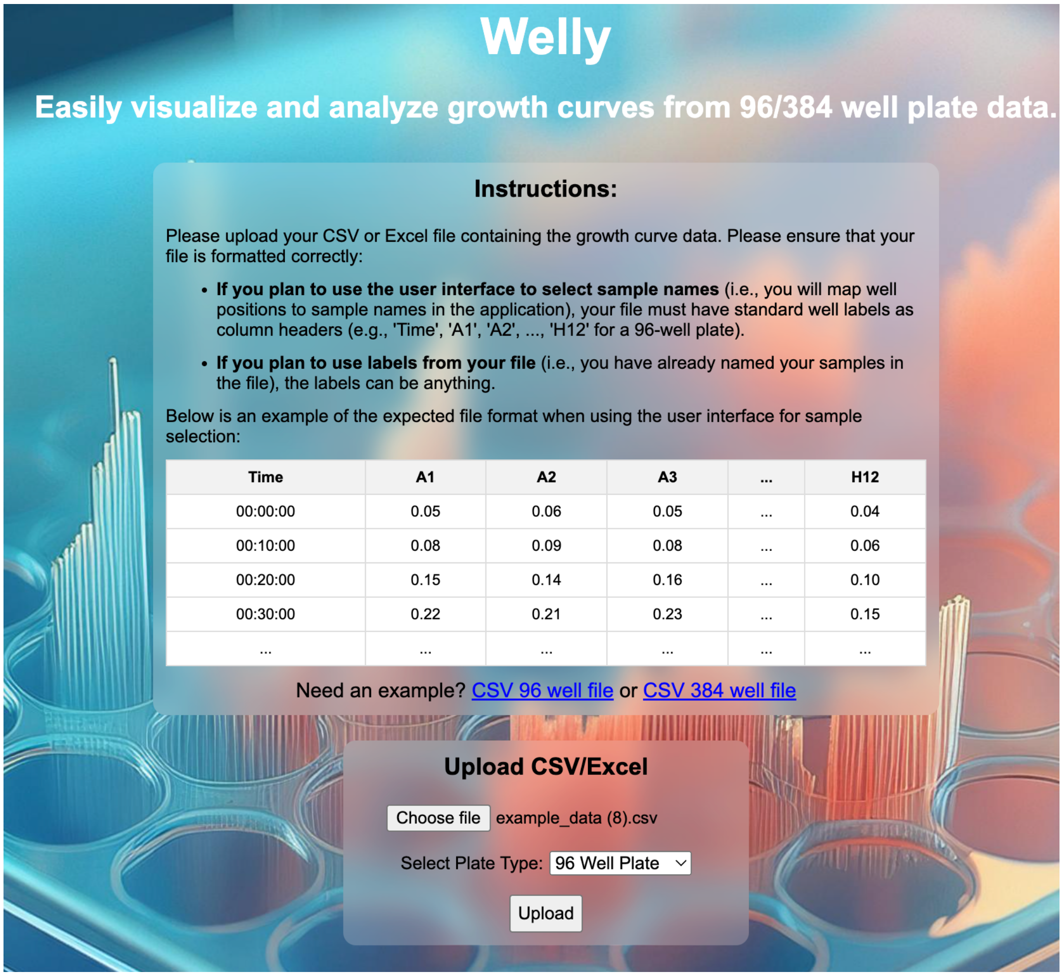

Step 2: Edit Well Plate Data

1. Enter a sample name in the text box.

2. Click wells to assign replicates. Same sample names will be averaged together.

3. Check 'Use CSV/Excel Labels' to retain labels from your file.

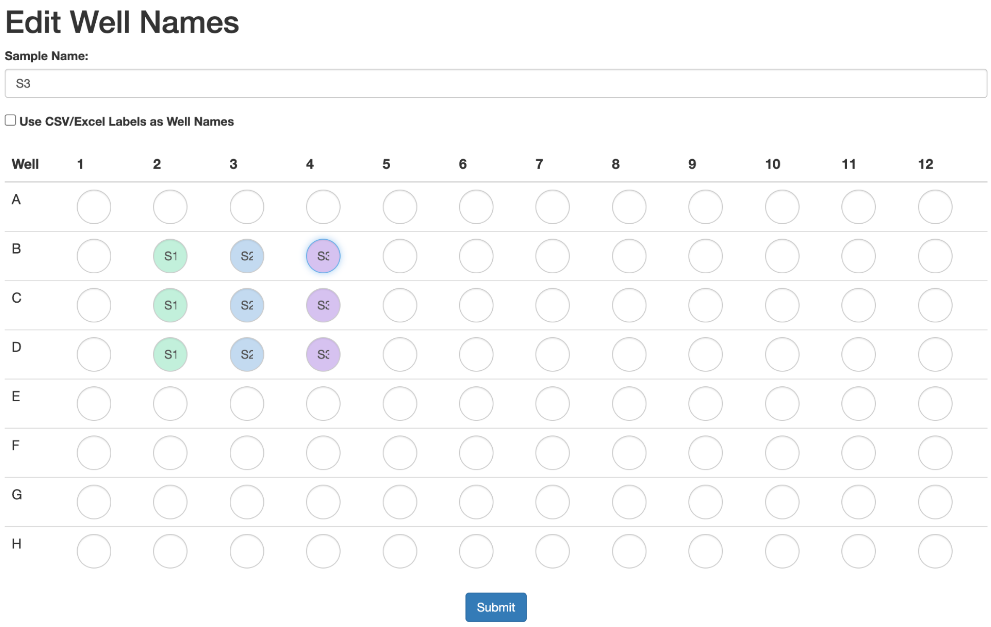

Step 3: Analyze Results

The system displays growth curves with mean OD and standard deviation.

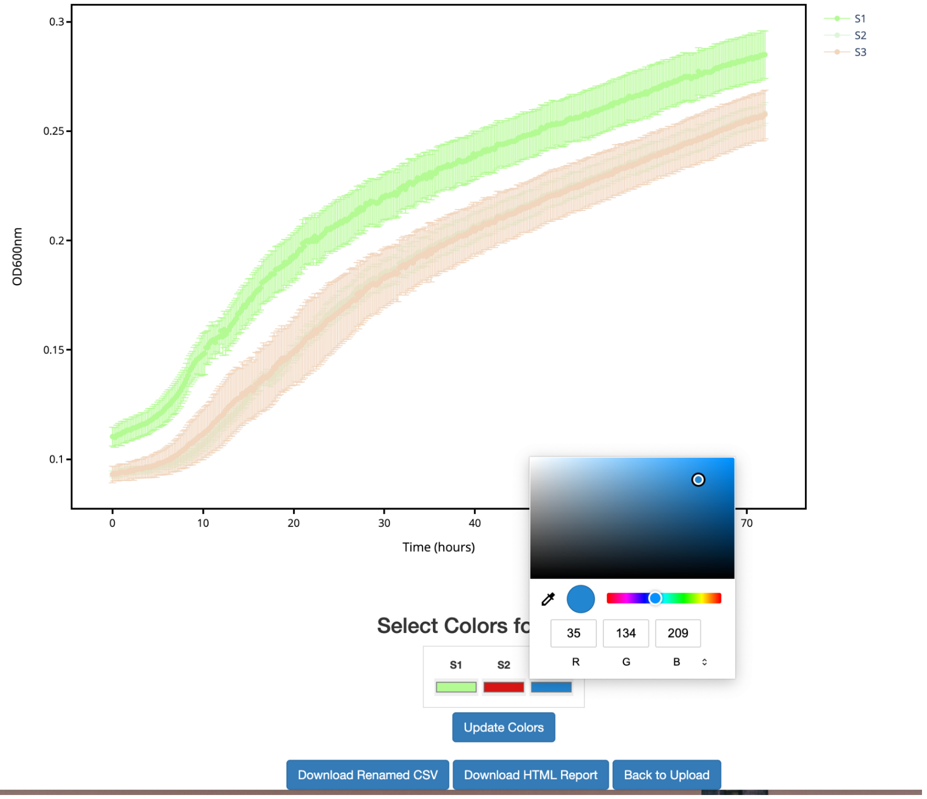

Custom Color Selection

Use the color picker to modify colors and click 'Update Colors' to refresh.

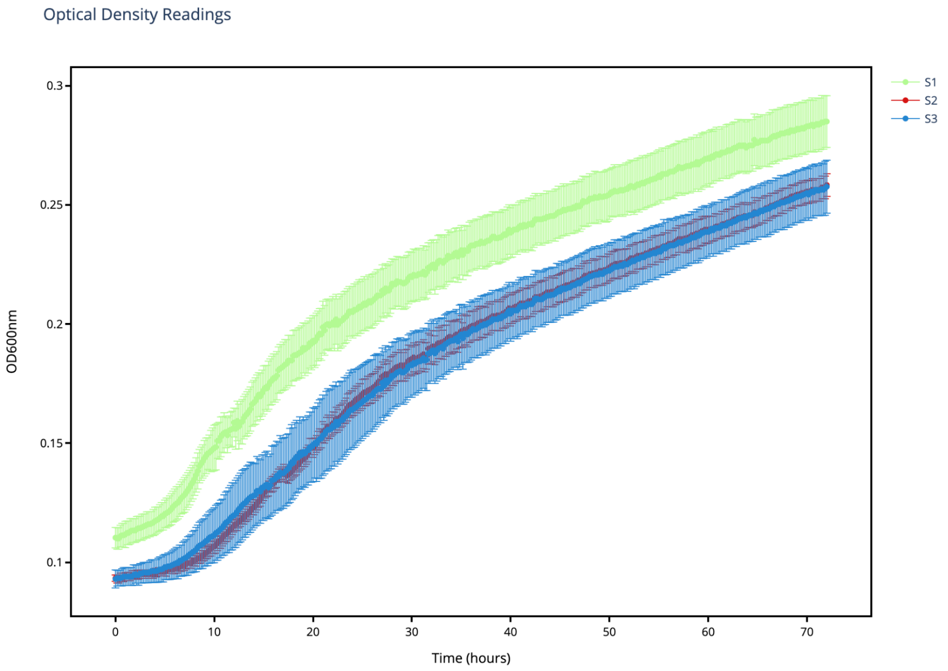

Step 4: Download Reports

Download the renamed dataset and HTML report for detailed analysis.

The report includes growth curves, heatmaps, and summary statistics.

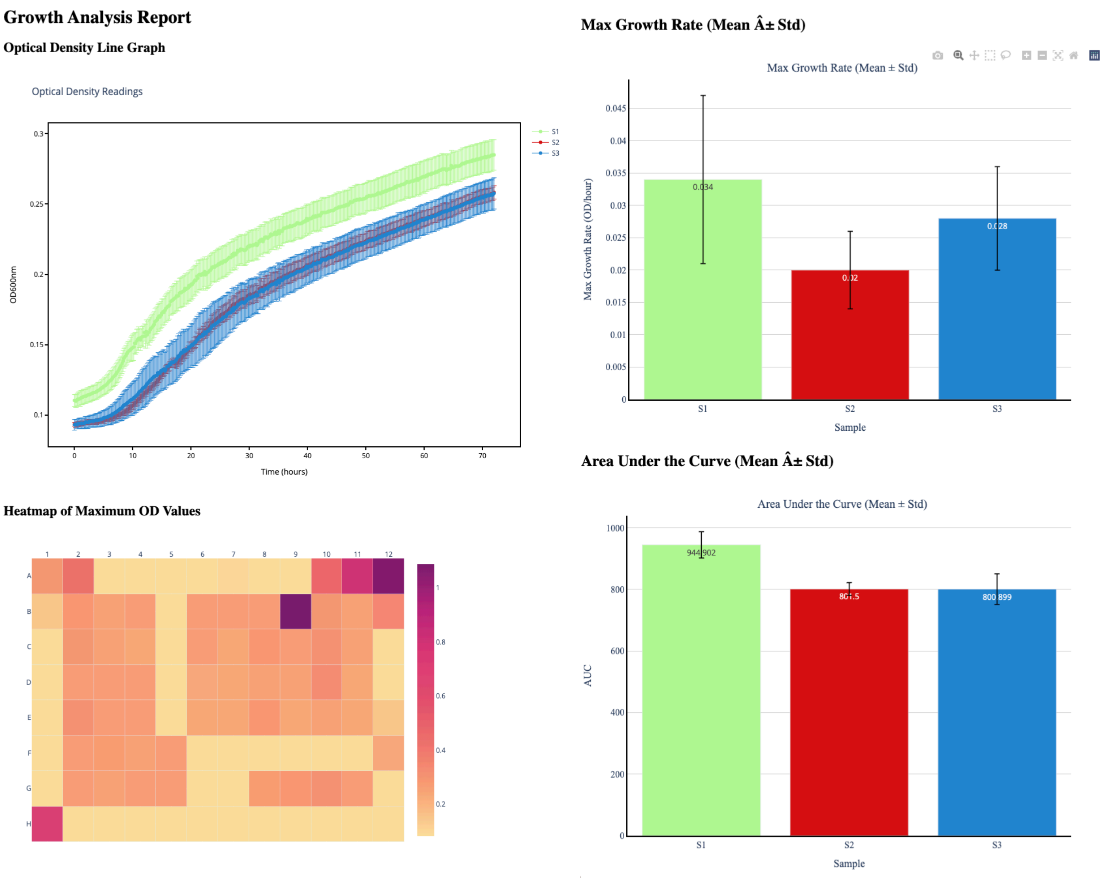

Definitions: Key Metrics

Maximum Growth Rate

The maximum growth rate is determined as the steepest slope of OD over time:

$$\text{Growth Rate} = \Delta OD / \Delta Time$$

Area Under the Curve (AUC)

The AUC quantifies cumulative growth over time and is calculated using the trapezoidal rule:

$$AUC = \Sigma \left((OD_{i+1} + OD_{i}) / 2 \right) * (Time_{i+1} - Time_{i})$$
